# Obesity and lung function: a systematic review

**DOI:** 10.1590/S1679-45082014RW2691

**Published:** 2014

**Authors:** Luciana Costa Melo, Maria Alayde Mendonça da Silva, Ana Carolina do Nascimento Calles

**Affiliations:** 1 Universidade Federal de Alagoas, Maceio, AL, Brazil

**Keywords:** Obesity, Physiological respiratory phenomena, Spirometry, Plethysmography, Review

## Abstract

Obesity is a chronic disease characterized by the excessive accumulation of body fat that is harmful to the individuals. Respiratory disorders are among the comorbidities associated with obesity. This study had the objective of investigating the alterations in respiratory function that affect obese individuals. A systematic review was performed, by selecting publications in the science databases MEDLINE and LILACS, using PubMed and SciELO. The articles that assessed pulmonary function by plethysmography and/or spirometry in obese individuals aged under 18 years were included. The results demonstrated that the obese individuals presented with a reduction in lung volume and capacity as compared to healthy individuals. Reduction of total lung capacity and reduction of forced vital capacity, accompanied by reduction of the forced expiratory volume after one second were the most representative findings in the samples. The articles analyzed proved the presence of a restrictive respiratory pattern associated with obesity.

## RESUMO

A obesidade é uma doença crônica, caracterizada pelo acúmulo excessivo de gordura corporal, que ocasiona prejuízos ao indivíduo. Dentre as comorbidades associadas à obesidade, estão as disfunções respiratórias. O presente estudo teve por objetivo investigar as alterações da função pulmonar que acometem indivíduos obesos. Foi realizada uma revisão sistemática, por meio da seleção de publicações nas bases científicas de dados MEDLINE e LILACS, por meio do PubMed e SciELO. Foram inclusos os trabalhos que avaliaram a função pulmonar, por meio de pletismografia e/ou espirometria, em indivíduos obesos maiores de 18 anos. Os resultados demonstraram que os indivíduos obesos apresentaram redução dos volumes e capacidades pulmonares quando comparados a indivíduos eutróficos. Redução da capacidade pulmonar total e redução da capacidade vital forçada, acompanhadas de redução do volume expiratório forçado após um segundo, foram os achados mais representativos dentre as amostras. Os trabalhos analisados comprovam a presença de padrão respiratório restritivo associado à obesidade.

## INTRODUCTION

Obesity is a chronic disease characterized by excessive accumulation of body fat that is harmful to individuals.^(1)^ According to the World Health Organization,^(2)^ obesity has reached epidemic proportions, affecting people of all ages and social classes in the world. The number of obese individuals has doubled since 1980, and in 2008, more than 1.4 billion adults were obese.^(2)^

Overweight promotes metabolic and structural changes that make the obese individual more susceptible to several events, including cardiovascular, pulmonary renal and biliary diseases, metabolic alterations, obstructive sleep apnea and some types of neoplasms.^(1,3,4)^

Over the last years, the repercussions of adiposity on respiratory function have been studied; however, there is no consensus as to the physiological mechanisms that lead to respiratory complications.^(5)^ It is known that adequate pulmonary function depends on the harmonic operation of the structures that compose the respiratory system. In obese individuals, structural changes of the thoracic-abdominal region lead to limited diaphragm mobility and rib movement, both essential for appropriate ventilatory mechanics. Additionally, the adipose tissue is an endocrine and paracrine organ that produces a large number of cytokines and bioactive mediators, thus generating in obese individuals, a pro-inflammatory state which is associated with hypodevelopment of the lungs, atopy, bronchial responsiveness, increased risk of asthma, and phenotypic modifications for this disease.^(6)^

One must point out that excess body fat is classified in categories according to its severity (mild, moderate, or severe) or the type of distribution of this fat (gynoid or android). In this way, there are several phenotypes of obesity.^(4)^ The studies published so far investigated the lung function of individuals with different degrees of obesity and assessed lung volumes and capacities in an individual manner. Therefore, a review is needed that pools all the results of these studies in a critical way, in order to identify which variables that evaluate lung function are impaired in obese individuals. From there, it should be possible to direct the appropriate care for the this population.

Within this context, the present study was developed by means of a systematic review of literature, with the objective of investigating changes in pulmonary capacities and volumes associated with obesity.

## METHODS

### Identification of the studies

To identify the articles available in the literature on the theme under study, the MEDLINE and LILACS databases were used and accessed through PubMed (http://www.pubmed.gov) and SciELO (http://www.scielo.br).

The following keywords were used in the search: in English, “obesity and lungfunction”, “obese and lung function”, and, in Portuguese, “*obesidade e função pulmonar*”. “Lung function” is not a keyword indexed in Mesh or Decs, but given the importance of this term for the search, it was adopted as a keyword. The investigation was limited to the presence of the keywords in the title and/or abstracts of the articles.

### Selection of the studies

The studies that evaluated lung function of obese or overweight individuals by means of spirometry or plethysmography tests were selected. Obese individuals were considered as those with a body mass index (BMI) >30kg/m^2^ and overweight people as those with BMI ≥25kg/m^2^.^(7)^ As variables for the lung function evaluations, we searched for lung volumes and capacities: total lung capacity (TLC), vital capacity (VC), forced vital capacity (FVC), residual functional capacity (RFC), forced expiratory volume after 1 second (FEV_1_), inspiratory reserve volume (IRV), tidal volume (TV), expiratory reserve volume (ERV), and residual volume (RV).

After identification of the keywords in the title and/or abstract, the selected articles went through reading of the abstracts to evaluate how appropriate the methods and study populations were as to the proposal of this review. Those tests that satisfied the predetermined eligibility criteria were acquired in their entirety for detailed analysis and data extraction.

The articles describing studies in children, which did not use spirometry or plethysmography to evaluate lung volumes, or that evaluated lung function of individuals with chronic lung disease, and review articles were excluded.

### Data extraction

The search and analysis of articles, as per the strategy mentioned above, were conducted independently by two evaluators (Author 1 and Author 2). Controversies as to eligibility of the publications to compose the database were resolved by consensus.

There was no blinding as to names of the authors, journals, or organizations. This is a possible source of bias, even if involuntary. On the other hand, as a strategy to minimize biases, each author involved in the selection of articles only acknowledged articles selected by the other author at the time of consensus for choosing appropriate studies for data extraction. Additionally, since the search was limited to the keywords chosen, it is possible that other studies on the theme were not taken into consideration.

### Data tabulation

The texts selected were read in detail to compile data corresponding to pulmonary function and overweight. Data relative to characterization of the samples, variables studied, and results obtained in the studies were organized in a Microsoft Office Excel 2007 spreadsheet. From that, it was possible to analyze the changes in values obtained by spirometry and/or plethysmography in obese individuals.

## RESULTS

Eighty-seven studies were found using the keywords “obese and lung function”; 144 with the keywords “obesity and lung function”; no studies were found in Portuguese with these keywords. According to the pre-established criteria, nine studies were selected (Charts 1 and 2).

**Chart 1:** General description of the studies selected

Authors	Location	Type of study	Sample
Carey et al.^(8)^	United Kingdom	Longitudinal before/after study	1,543 men and1,848 women, aged 18 to 73 years
Zerah et al.^(9)^	France	Cross-sectional study	15 men and 31 women, 18 to 63 years, divided into 3 groups: G1: overweight (13) G2: obesity grade I and II (24) G3: morbid obesity(9)
Jones e Nzekwu^(10)^	Canada	Cross-sectional study	158 men and 215 women over 18 years were divided into 5 groups: G1: normal weight (93) G2: overweight (78) G3: mild obesity (92) G4: moderate obesity (67) G5: morbid obesity (43)
Thyagarajan et al.^(11)^	United States	Cohort study	2,191 men and 2,543 women, white and black, aged between 18 and 30 years
Ceylan et al.^(12)^	Turkey	Cross-sectional study	31 women and 22 men aged between 18 and 66 years
Gabrielsen et al.^(13)^	Norway	Cross-sectional study	35 men and 114 women (morbidly obese), with an mean age of 43 years, descendants of Europeans
Steele et al.^(14)^	United Kingdom	Cohort study	120 men (mean age 40.2 years) and 200 women (mean age 40.8 years). Of the total number of subjects, 32% presented with normal weight, 40% were overweight and 28% were obese
Canoy et al.^(15)^	United Kingdom	Cohort study	9,674 men and 1,1876 women aged between 45 and 79 years

All studies used the classification of obesity of the World Health Organization.^(7)^

**Chart 2:** Reduced pulmonary volumes and capacities in obese individuals of the studies analyzed

Authors	TLC	VC	FVC	RFC	FEV_1_	ERV	RV
Carey et al.^(8)^					# *		
Zerah et al.^(9)^	# *	# *		# *	# *	# *	
Jones e Nzekwu^(10)^	# *	# *	#	# *	#	# *	# *
Thyagarajan et al.^(11)^			# *		# *		
Ceylan et al.^(12)^			#	# *	#	# *	
Gabrielsen et al.^(13)^		#	#		#	# *	
Steele et al.^(14)^			# *		# *		
Canoy et al.^(15)^			# *		# *		
Saliman et al.^(16)^	# *	#	# *	#	# *	#	#

TLC: total lung capacity; VC: vital capacity; FVC: forced vital capacity; RFC: residual functional capacity, FEV_1_: forced expiratory volume after 1 second; ERV: expiratory reserve volume; RV: residual volume.

# Variables analyzed by the studies (as per the articles published);

* variables with a reduction associated with obesity.

Of the articles selected, five came from cross-sectional studies and four from cohort studies. Two studies were carried out in the United States, six in European countries, and one in Turkey. All studies had samples composed by both genders, with a predominance of females. As to the lung function evaluation, two articles used plethysmography,^(9,10)^ six used spirometry,^(8,11–15)^ and one used both tests.^(16)^

Decreased FEV_1_, associated with overweight, was found in 6 studies.^(8,9,11,14–16)^ Carey et al. identified that a 10kg weight gain induced a drop in FEV_1_ by 96mL in men and by 51mL in women.^(8)^ Thyagarajan et al. observed that individuals with a BMI ≥26.4kg/m^2^ had a reduction of 64mL in FEV_1_ in 10 years. During the same period, individuals with a BMI <21.3kg/m^2^ showed an increase of 60mL in the FEV_1_, with no reduction up to 38 years of age.^(11)^ The decrease in FEV_1_ in this study was more expressive in obese individuals, who had a greater weight gain during the study. Corroborating these findings, in evaluating 136 morbid obese individuals, Saliman et al. found that women had a mean FEV_1_ of 79% of that predicted, and men had 65% of the predicted value.^(16)^ Although the studies point towards a decrease of FEV_1_ in men and women, Carey et al. and Steele et al. demonstrated that central fat deposit seems to have a stronger association with breathing mechanisms in men compared to women.^(8,14)^

Four studies found an inverse relation between FVC and obesity.^(11,14–16)^ In one cohort studied by Thyagarajan et al., in 10 years, participants with BMI ≥26.4kg/m^2^ presented with a decrease by 185mL in the FVC, while those with BMI <21.3kg/m^2^ showed an average increment of 71mL. Individuals that gained more weight had a greater drop in FVC.^(11)^ In the morbid obese from the study by Saliman et al. the mean value of was 83% of that predicted in women and 71% of the predicted in men.^(16)^ Just as for FEV_1_, Steele et al. found that the central fat deposit seemed to be more strongly related to the lung function in men than in women.^(14)^

As to TLC, three articles demonstrated a decreased association with overweight.^(9,10,16)^ Saliman et al. found a mean value of TLC of 87% and 82% of that predicted in women and men, respectively. In the study, TLC and BMI showed an inversely proportional relationship.^(16)^ In the study by Jones and Nzekwu, individuals with normal weight and overweight showed no significant difference as to TLC. However, individuals with normal weight had a significantly greater TLC when compared to those with mild, moderate, and morbid obesity.^(10)^

RFC and ERV were decreased in the samples of four studies.^(9,10,12,13)^ In Jones and Nzekwu, the group with normal weight had significantly different values of RFC and ERV relative to the other groups.^(10)^ But the mild, moderate, and morbid obese show no statistically significant differences among themselves. Still in this study, among the variables studied, RFC and ERV had the most expressive values, decreasing with the increase of BMI. Confirming these findings, the most common abnormality found by Ceylan et al.^(12)^ in the individuals with overweight and obesity was a reduction in RFC and ERV, both in men and in women.

VC also suffered influence from overweight, decreasing with the increase of obesity.^(9,10)^ Just as for TLC, the study by Jones and Nzekwu^(10)^ did not demonstrate a difference as to VC among people with normal weight and overweight, but between the normal weight and the obese, there was a significant difference. On the other hand, Gabrielsen et al. found no association between alterations of VC and BMI, but they noted that the neck circumference had a negative correlation with VC.^(13)^

Jones and Nzekwu, further found a decrease in VR in obese individuals when compared to normal-weight individuals.^(10)^

## DISCUSSION

Breathing is an essential function for survival, and alterations in lung function can hinder quality of life and performance in activities of daily living. To maintain respiratory homeostasis, the structures that compose the respiratory system need to work in equilibrium, that is, the lungs should be ventilated and the gases should diffuse through the alveolar-capillary barrier.^(17)^

An efficient form to evaluate lung function is by determining lung volumes, which offer information essential for the characterization of the pathophysiological state resulting from abnormalities in the pulmonary-ventilatory processes.^(18)^ The most accurate techniques for determining lung volumes are spirometry and plethysmography.^(19)^ Castrejón Vázquez et al.^(20)^ carried out a comparative study between the two techniques and observed that both provided similar results, despite the fact that plethysmography shows greater sensitivity. In this way, even if there are articles using different techniques, it is possible to compare them without compromising the results.

The reduction of FVC happens both in restrictive and obstructive diseases, but the percentage of reduction of FEV_1_ accompanies the reduction of FVC.^(21)^ Such a situation was found in the studies that evaluated these two variables,^(11,14–16)^ suggesting a restrictive pattern in the samples studied. Since the subjects evaluated did not have respiratory disease, the restriction could be explained by the alteration in ventilatory mechanics experienced by the obese. In normal breathing, the diaphragm contracts, pushing the abdominal content downward and forward, and at the same time, contraction of the external intercostal muscles tractions the ribs upwards and forward.^(17)^ In obese individuals, this mechanism is hindered, since the excess adiposity that covers the thorax and the abdomen encumbers the breathing muscles. Such a mechanism also explains the findings by Steele et al.^(14)^ that demonstrate a stronger relation of the FVC and FEV_1_ alterations in men when compared to women. This is because the male gender has a greater frequency of android fat deposit pattern, while gynoid fat is more common in the female gender.^(22)^ In other words, a greater deposit of fat in the abdominal region generates a greater resistance to diaphragmatic contraction, hindering ventilatory mechanics.

In individuals with no obstructive disease, FVC is normally equal to the VC.^(21)^ Due to lack of data on these two variables in the studies selected, it was not possible to assess a negative relation between obesity and VC,^(9,10)^ which is justified by the same physiological mechanisms that explain the reduction of FVC. It is important to point out that the anthropometric measurement used can interfere in the presence of an association between obesity and VC, as demonstrated by Gabrielsen et al.^(13)^ Nevertheless, more studies are necessary to evaluate that aspect.

Under physiological conditions, RFC represents the equilibrium point of elastic retractions between the lungs and the chest wall.^(18)^ Therefore, conditions that limit chest expansibility will invariably affect the volume contained in the lungs at the end of a spontaneous expiration (RFC). RFC is understood as the sum of VR and ERV. Consequently, variations of the VR or ERV imply alterations of RFC, as demonstrated by Zerah et al.,^(9)^ Jones and Nzekwu,^(10)^ Ceylan et al.,^(12)^ and Gabrielsen et al.,^(13)^ in which both RFC and ERV were diminished. Nevertheless, Barreto pointed out that the reduction of RFC, as in obesity and in pregnancy, should be considered, but in and of itself it does not represent a restrictive disease.^(18)^

Since only three studies used plethysmography to evaluate lung function, the assessment of TLC was limited to these two articles, as spirometry is not capable of evaluating such a parameter.^(21)^ TLC is the volume contained in the lungs after a full inspiration and covers all the lung volumes.^(18)^ In this way, alterations in the other lung volumes compromise TLC. Thus, it is possible that more samples had a lower TLC, but that this was not diagnosed due to methodological limitation. According to Barreto,^(18)^ “TLC is the only lung volume with an absolute significance for the definition of the pathophysiological pattern: the presence of a restrictive abnormality is expressed by a TLC under the lower limit foreseen.”

Any interference in the chest bellows or in chest mobility, generating a decrease in lung volumes, can be considered a restrictive illness.^(21)^ In the obese, excess fat in the abdominal cavity and chest limit the two primary inspiratory movements: diaphragm contraction propelling the abdominal content downwards and forwards, and increased chest diameter by means of rib movement.^(17,23)^ The results of the studies evaluated in our review confirm the presence of a restrictive pattern in obese individuals when they demonstrated a decrease in lung volumes and capacities.

On the other hand, it is important to point out the existence of extremely obese individuals with normal spirometry, lung volume, and blood gas results.^(16)^ It is noteworthy, therefore, that the mechanisms that lead to lung function disorders in obese individuals are still not completely clarified, and further studies are required in this area.

The present review was limited to identifying the alterations in lung function present in obese individuals, without identifying the degree of obesity from which such modifications stem. Additionally, some studies did not evaluate all lung volumes and capacities, and lung function disorders could not be identified. For the results indicated by this review to be confirmed, random clinical trials must be conducted with the objective of identifying the repercussions of overweight for lung function.

Despite the previously mentioned limitations, the results highlighted by this review are a fruit of critical and systematic analysis of articles published in journals of renowned scientific quality. In this way, it furnished scientific subsidies for the appropriate management of the population investigated, besides pointing out the gaps that exist in literature so they can be filled by further studies.

## CONCLUSION

The obese individuals demonstrated reduced lung volumes and capacities when compared to normal-weight individuals. Reduction in total lung capacity and forced vital capacity, accompanied by reduced forced expiratory volume after one second were the most representative findings among the samples, both suggesting the presence of a restrictive respiratory pattern associated with obesity. Consequently, it is necessary to implement healthcare programs for this population, with the purpose of improving lung function and therefore improving quality of life of obese individuals. Although the investigations demonstrated the presence of lung alterations in the obese population, the physiological mechanisms that lead to such a situation are still not clear. Therefore, we suggest that more studies be carried out with the objective of clarifying them.
